# Promotion of autophagosome–lysosome fusion *via* salvianolic acid A-mediated SIRT1 up-regulation ameliorates alcoholic liver disease†

**DOI:** 10.1039/c8ra00798e

**Published:** 2018-06-05

**Authors:** Xue Shi, Ruimin Sun, Yan Zhao, Rong Fu, Ruiwen Wang, Huanyu Zhao, Zhecheng Wang, Fan Tang, Ning Zhang, Xiaofeng Tian, Jihong Yao

**Affiliations:** Department of Pharmacology, Dalian Medical University Dalian 116044 China yaojihong65@hotmail.com +86-0411-86110010 +86-0411-8611-0410; Department of Pharmacy, First Affiliated Hospital of Xinxiang Medical University Xinxiang 453100 China; Department of Pharmacy, Second Hospital of Dalian Medical University Dalian 116027 China; Department of General Surgery, Second Affiliated Hospital, Dalian Medical University Dalian 116023 China

## Abstract

Salvianolic acid A (SalA) is a water-soluble phenolic carboxylic acid extracted from *Salvia miltiorrhiza* that has extensive pharmacological activities and plays an essential role in liver disease treatment. However, the mechanism of SalA in treating alcoholic liver disease (ALD) remains unclear. Here, we studied the protective effects of SalA on chronic ethanol-induced liver injury involving Sirtuin 1 (SIRT1)-mediated autophagy activation. The results showed that SalA pretreatment reduced the levels of alanine aminotransferase (ALT), aspartate aminotransferase (AST), triglyceride (TG) and cholesterol (TC) *in vivo* and enhanced hepatic cell viability while mitigating apoptosis and hepatic steatosis *in vitro*. Furthermore, SalA protected against chronic ethanol-induced liver injury by restoring autophagosome–lysosome fusion, as indicated by the increased expression levels of LC3-II, cathepsin B, lysosomal-associated membrane protein 2 (LAMP-2), and RAB7 and the decreased expression of SQSTM1. More importantly, pretreatment with SalA significantly up-regulated the expression of SIRT1, an NAD^+^-dependent deacetylase. These increased levels of SIRT1 stimulated autophagy under conditions of chronic ethanol exposure. Interestingly, SIRT1 siRNA abrogated SalA-induced autophagosome–lysosome fusion. This finding indicates that the protective effects of SalA are associated with SIRT1 activation. Collectively, our study demonstrates that SalA pretreatment protects against chronic ethanol-induced liver injury *via* the SIRT1-mediated restoration of autophagosome–lysosome fusion.

## Abstract 2

Autophagosome and lysosome fusion was restored by salvianolic acid A-mediated SIRT1 up-regulation and protected against chronic ethanol-induced liver injury.

## Graphical abstract

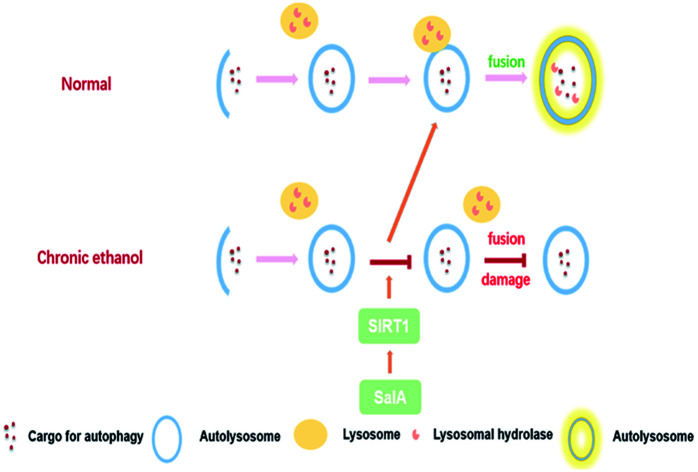

## Introduction

Alcoholic liver disease (ALD) due to excess alcohol consumption is a major cause of morbidity and mortality worldwide.^1^ ALD is a complex condition that involves a wide spectrum of hepatic lesions, including simple steatosis, alcoholic hepatitis, hepatic fibrosis, and cirrhosis.^2–4^ Chronic ethanol consumption causes dramatic metabolic changes, lipid homeostasis disruption, mitochondrial damage, reactive oxygen species (ROS) accumulation, and hepatocyte cell death; these metabolic changes accelerate the hepatotoxicity of ethanol and ultimately cause liver injury.^5–8^ Although much progress has been made in the past few decades, effective approaches for regulating ALD progression remain limited.

*Salvia miltiorrhiza*, commonly called “Danshen”, is an important medicinal plant that has been used in China for thousands of years. Salvianolic acid A (SalA) is one of the primary active, water-soluble components of *Salvia miltiorrhiza*.^9^ SalA has extensive bioactivities, including antioxidative, anti-inflammatory, anticirrhotic effects.^10^ SalA can prevent a variety of diseases, including coronary artery diseases, cerebral ischemia, and diabetes mellitus.^11–13^*Salvia miltiorrhiza* Bunge and its active component cryptotanshinone protect hepatocytes from acute ethanol-induced cytotoxicity and lipid accumulation.^14^ In addition, we have demonstrated that SalA has hepatoprotective effects against high-fat diet (HFD)-induced nonalcoholic fatty liver disease and concanavalin A-induced liver injury.^15,16^ However, the effects of SalA on ALD and potential molecular mechanisms remain unknown.

Autophagy is a self-degradation process in which dysfunctional or damaged components are incorporated into double-membrane vesicles called autophagic vacuoles (AVs) or autophagosomes, which fuse with lysosomes to be digested by resident hydrolases; this entire process is called autophagy flux.^17,18^ In addition, AV fusion with lysosomes to form autolysosomes is a crucial step in maintaining proper autophagy flux.^19,20^ A recent study shows that autophagy is important in limiting ethanol-induced liver injury and hepatocyte apoptosis because it removes damaged mitochondria and lipid droplets that have accumulated.^21^ Chronic alcohol consumption can suppress autophagy because alcohol damages the lysosomes, suppresses lysosomal proteolysis activity, and blocks autolysosome formation.^22,23^ Furthermore, the pharmacological modulation of autophagy in the liver can alleviate hepatic steatosis and liver injury caused by excessive alcohol intake; this information suggests that autophagy may be a potential therapeutic target for treating ALD.^24^ Therefore, we investigated whether autophagy is involved in the protective role of SalA in ALD.

Sirtuin 1 (SIRT1) is currently the most studied member of the sirtuin family; SIRT1 is involved in many biological processes, including lipid metabolism, oxidation, inflammation, and apoptosis.^25–28^ We have demonstrated that SIRT1 participates in the alleviation of chronic alcoholic liver injury by preventing fat accumulation, decreasing ROS production, and reducing inflammation and cell death.^29,30^ Interestingly, SIRT1 deacetylates a number of transcription factors, including histone H4, NF-κB, and p53.^31,32^ The SIRT1/FoxO pathway accelerates AV development, augments autolysosome formation, and further stimulates autophagy flux.^33^ These effects suggest that SIRT1 may be a positive regulator of autophagy.

In this study, we investigated the effects of SalA on chronic ethanol-induced liver injury and assessed the involvement of the SIRT1/autophagy pathway. The present study may offer new insight into chronic ethanol-induced liver injury treatment and provide diverse therapeutic targets for treating ALD.

## Materials and methods

### Reagents

SalA (>98% purity) was purchased from Shanghai Winherb Medical Science Co., Ltd. (Shanghai, China) and dissolved in pathogen-free saline. Ethanol (>99% purity) was obtained from Sigma Co., Ltd. (St. Louis, MO, USA).

### Animals and treatments

Male Sprague-Dawley rats weighing 180 to 220 g were obtained from the Experimental Animal Center of Dalian Medical University (Dalian, China). All animal procedures were conducted according to the guidelines of the Institutional Animal Care and Use Committee of Dalian Medical University and experiments were approved by the Institutional Ethics Committee of Dalian Medical University. Sixty rats were maintained under standard laboratory conditions for one week. Then, the rats were randomly divided into five groups: (1) normal nonethanol diet (ND); (2) ND + SalA (16 mg kg^−1^ d^−1^); (3) Lieber–DeCarli ethanol diet (ED); (4) ED + SalA (8 mg kg^−1^ d^−1^), and (5) ED + SalA (16 mg kg^−1^ d^−1^). Rats were fed a Lieber–DeCarli ethanol diet or a normal nonethanol diet as previously described;^34^ these diets contained the same number of calories. The rats were intragastrically administered SalA (8 or 16 mg kg^−1^ d^−1^) or the same volume of normal saline for 8 weeks.

### Measurement of serum levels of alanine aminotransferase (ALT), aspartate aminotransferase (AST), triglyceride (TG), and cholesterol (TC)

Rat blood samples were acquited from the abdominal aorta and centrifuged at 3000 rpm for 15 min to separate serum. The serum levels of ALT, AST, TG and TC were measured using enzymatic assay kits according to the manufacturer's instructions (Nanjing Jiancheng Corp., Nanjing, China).

### Liver histopathologic examination

Liver tissues were fixed, paraffin-embedded, and sliced into 5 μm sections. Then, the slides were stained with hematoxylin and eosin (H&E) and examined under a light microscope.

### Cell culture and cell viability assay

The mouse AML-12 hepatocyte cell line was purchased from the American Type Culture Collection (ATCC, USA). AML-12 cells were cultured in 90% 1 : 1 Dulbecco's modified Eagle's medium : Ham's F12 medium (DMEM : F12) with 1% insulin–transferrin–selenium (ITS) (Gibco, CA, USA), 40 ng mL^−1^ dexamethasone (Gibco, CA, USA), and 10% (v/v) fetal bovine serum (FBS) (Gibco, CA, USA). The cells were maintained in a humidified incubator with 5% CO_2_ at 37 °C. To determine cell survival rates, CCK-8 assays (Selleck Chemicals, Shanghai, China) were used according to the manufacturer's instructions.

### Transmission electron microscopy (TEM)

Samples were fixed in a 0.1 M sodium cacodylate buffer (pH 7.4) and 2.5% glutaraldehyde solution for 4 h. Then, the samples were rinsed (three times, 10 min) with 0.1 M sodium cacodylate buffer (pH 7.4) and 7.5% sucrose and postfixed in a 1% OsO_4_ solution for 1 h. After dehydration using an ethanol gradient (70% ethanol (20 min), 96% ethanol (20 min), and 100% ethanol (two times, 20 min)), the samples were embedded in Durcupan ACM. Ultrathin sections were stained with uranyl acetate and lead citrate. Sections were examined under a JEOL JEM-2000EX microscope (JEOL, Tokyo, Japan) at 60 kV.

### Terminal deoxynucleotidyl transferase-mediated dUTP nick end labeling (TUNEL) assay

TUNEL staining was performed using an *In Situ* Cell Death Detection Kit (Roche, NJ, USA) according to the manufacturer's instructions. DAPI solution was used to stain the nuclei.^35^

### Nile red staining

Nile red is a selective fluorescent stain for intracellular lipid droplets. AML-12 cells were fixed in 4% paraformaldehyde and stained with Nile red solution (1 μg mL^−1^) (Sigma, no. 19 123) at 37 °C for 10 min in the dark. Lipid-bound Nile red was observed under a fluorescence microscope.

### Western blotting analysis

Total proteins were extracted from liver tissues by a protein extraction kit (KeyGen Biotech, Nanjing, China), and cultured cells were lysed in RIPA buffer according to the manufacturer's instructions. Protein concentrations were determined using a BCA protein assay kit (Beyotime Biotech, Shanghai, China). The same amount of protein from each sample was separated by 10–15% SDS-PAGE (Bio-Rad, Hercules, CA, USA). Blots were incubated overnight at 4 °C with the following primary antibodies: SIRT1 (Abcam Ltd, Cambridge, UK); LC3, SQSTM1, lysosomal-associated membrane protein 2 (LAMP-2), and RAB7 (Bioworld Technology, Inc., St Louis Park, MN, USA); and β-actin and cathepsin B (Proteintech Group Inc., Wuhan, China). After subsequent washing with TTBS, the blots were immunostained with secondary antibodies at 37 °C for 2 h. The membranes were exposed to Enhanced Chemiluminescence Plus reagent (Beyotime Institute of Biotechnology, Shanghai, China). Images were captured by a BioSpectrum 410 multispectral imaging system with a Chemi 410 HR camera and analyzed with Gel-Pro Analyzer Version 4.0 (Media Cybernetics, MD, USA).

### RNA interference

AML-12 cells were transfected with specific siRNA or nonbinding control siRNA (100 nM) using Lipofectamine 3000 (Invitrogen, Karlsruhe, Germany) according to the manufacturer's instructions. The small interfering RNA (siRNA) sequence targeting SIRT1 was as follows: sense 5′-CCCUGUAAAGCUUUCAGAAdtdt-3′ and antisense 5′-UUCUGAAAGCUUUACAGGGdtdt-3′. Both specific and control siRNAs were obtained from Genepharma (Shanghai, China). After siRNA transfection for 48 h, the cells were treated with 50 μM SalA for an additional 6 h. Then, the proteins were extracted for western blotting.

### Adenoviral vectors

AML-12 cells were plated in 6-well plates and allowed to reach 50–70% confluence for transfection. MRFP-GFP-LC3 adenoviral vectors were purchased from HanBio Technology (Shanghai, China), and the adenoviral infection was performed according to the manufacturer's instructions. In the green and red merged images, the red and yellow puncta represent autolysosomes and autophagosomes, respectively. LC3 puncta were examined with a Leica TCS SP5 II confocal microscope (Leica, Wetzlar, Germany).

## Results

### SalA protects against chronic ethanol-induced liver injury

**Fig. 1:**
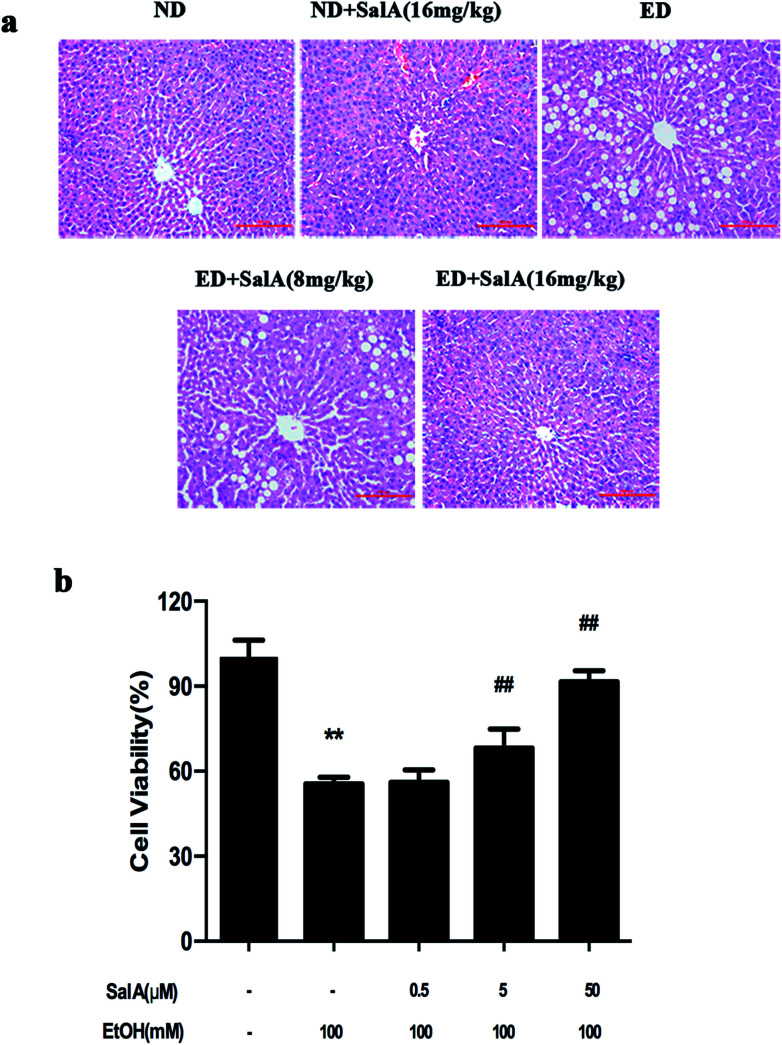
SalA pretreatment attenuates chronic ethanol-induced liver injury. (a) H&E staining. The experimental groups subjected to H&E staining were as follows: ND; ND + SalA (16 mg kg^−1^); ED; ED + SalA (8 mg kg^−1^); and ED + SalA (16 mg kg^−1^). H&E-stained sections were photographed at 200× magnification. (b) Cell viability. AML-12 cells were pretreated with SalA (0.5, 5 or 50 μM) for 6 h and then treated with ethanol for 24 h. Cell viability was assessed using CCK8 assays. The data are presented as the mean ± S.D. (*n* ≥ 8). **P < 0.01 *versus* the control group, ^##^*P* < 0.01 *versus* the ethanol group.

First, we evaluated whether SalA could protect rats against chronic ethanol-induced liver injury. After 8 weeks, the ALT, AST, TC, and TG levels were clearly higher in the rats fed a diet with ethanol compared with the ND group (Table 1). However, compared to ethanol treatment alone, SalA pretreatment abrogated these increases in ALT, AST, TG, and TC levels. Liver injury caused by chronic ethanol consumption and the protective effects of SalA were further evaluated by histological assays. As shown in Fig. 1a, chronic ethanol administration caused the formation of a large number of fat droplets, increased cellular size, and induced hepatocellular macrovesicular steatosis in the liver; these effects were significantly reduced by pretreatment with SalA. *In vitro*, SalA pretreatment (0.5, 5, or 50 μM) increased the cell viability, which inhibited by ethanol consumption, in a dose-dependent manner (Fig. 1b). Furthermore, the ethanol-induced increase in ALT, AST, TC, and TG levels was significantly prevented by SalA pretreatment in AML-12 cells (Table 2). These results indicate that SalA effectively protected against chronic ethanol-induced liver injury *in vivo* and *in vitro*.

**Table 1:** SalA regulates the serum levels of alanine aminotransferase (ALT), aspartate aminotransferase (AST), triglyceride (TG) and cholesterol (TC) in the ALD rats^a^

Group	ALT (IU L^−1^)	AST (IU L^−1^)	TC (mmol L^−1^)	TG (mmol L^−1^)
ND	9.69 ± 1.14	18.74 ± 3.36	2.12 ± 0.15	0.99 ± 0.12
ND + SalA (16 mg kg^−1^)	9.41 ± 1.11	18.66 ± 1.88	2.03 ± 0.17	1.02 ± 0.19
ED	15.13 ± 2.17**	44.08 ± 8.99**	3.00 ± 0.30**	2.16 ± 0.44**
ED + SalA (8 mg kg^−1^)	12.31 ± 1.63^##^	30.12 ± 9.43^##^	2.66 ± 0.33^&^	1.56 ± 0.37^##^
ED + SalA (16 mg k^−1^)	10.72 ± 1.33^##^	23.51 ± 4.90^##^	2.22 ± 0.16^##^	1.38 ± 0.29^##^

a The data are presented as the mean ± S.D. (*n* = 6). **p < 0.01 *versus* ND group, ^&^*p* < 0.05 *versus* ED group, ^##^*p* < 0.01 versus ED group.

**Table 2:** SalA pretreatment protects against chronic ethanol-induced AML-12 cell injury^a^

Group	ALT (IU L^−1^)	AST (IU L^−1^)	TC (mmol L^−1^)	TG (mmol L^−1^)
Control	20.59 ± 4.65	21.93 ± 2.02	54.15 ± 4.59	43.44 ± 5.11
Control + SalA (50 μM)	18.84 ± 2.33	20.58 ± 2.34	50.19 ± 2.24	38.10 ± 2.53
Ethanol (100 mM)	42.57 ± 6.61**	47.01 ± 4.48**	135.98 ± 6.50**	121.98 ± 6.24**
Ethanol + SalA (50 μM)	26.97 ± 0.94^##^	28.93 ± 2.87^##^	72.36 ± 10.89^##^	59.03 ± 5.26^##^

a The data are presented as the mean ± S.D. (*n* = 6). **p < 0.01 *versus* control group. ^##^*p* < 0.01 *versus* ethanol group.

### SalA prevents chronic ethanol-induced liver injury by activating autophagy

**Fig. 2:**
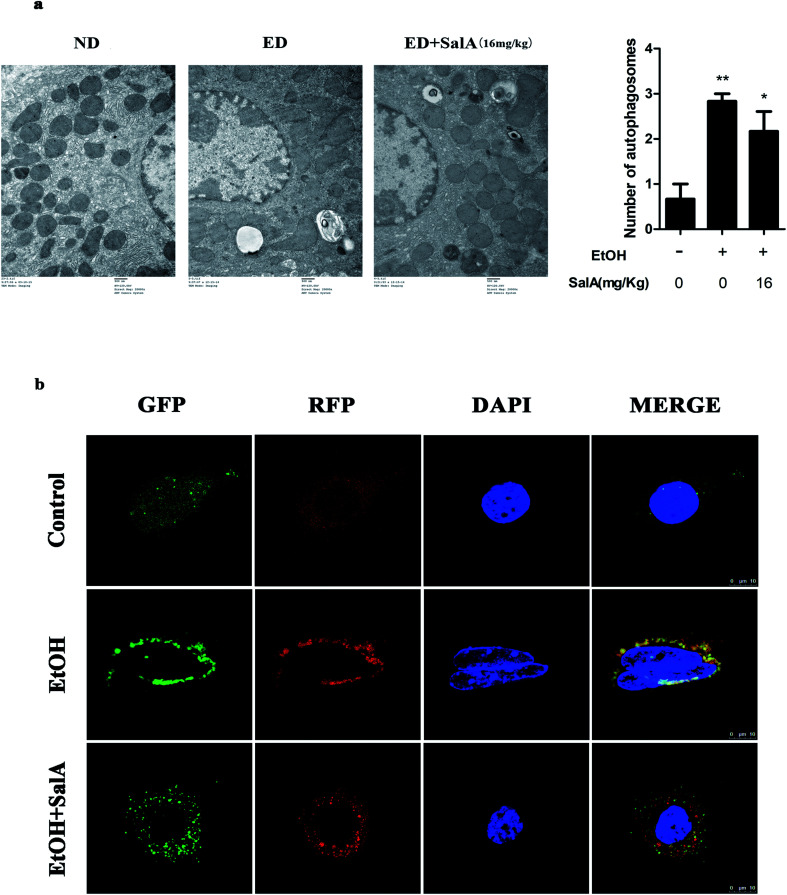
SalA mediates autophagy activation. (a) Effects of SalA on AV formation in ALD liver were assessed using the TEM assay (1500×), and autophagosomes were quantified. The data are presented as the mean ± S.D. *P < 0.05 *versus* the ND group, **P < 0.01 *versus* the ND group (*n* = 3). (b) Autophagy flux visualized through confocal microscopy. AML-12 cells were infected with adenoviruses as described in Materials and methods. Autophagy flux was increased when the numbers of both yellow and red puncta were increased in cells, whereas autophagy flux was blocked when the number of only yellow puncta was increased in AML-12 cells.

**Fig. 3:**
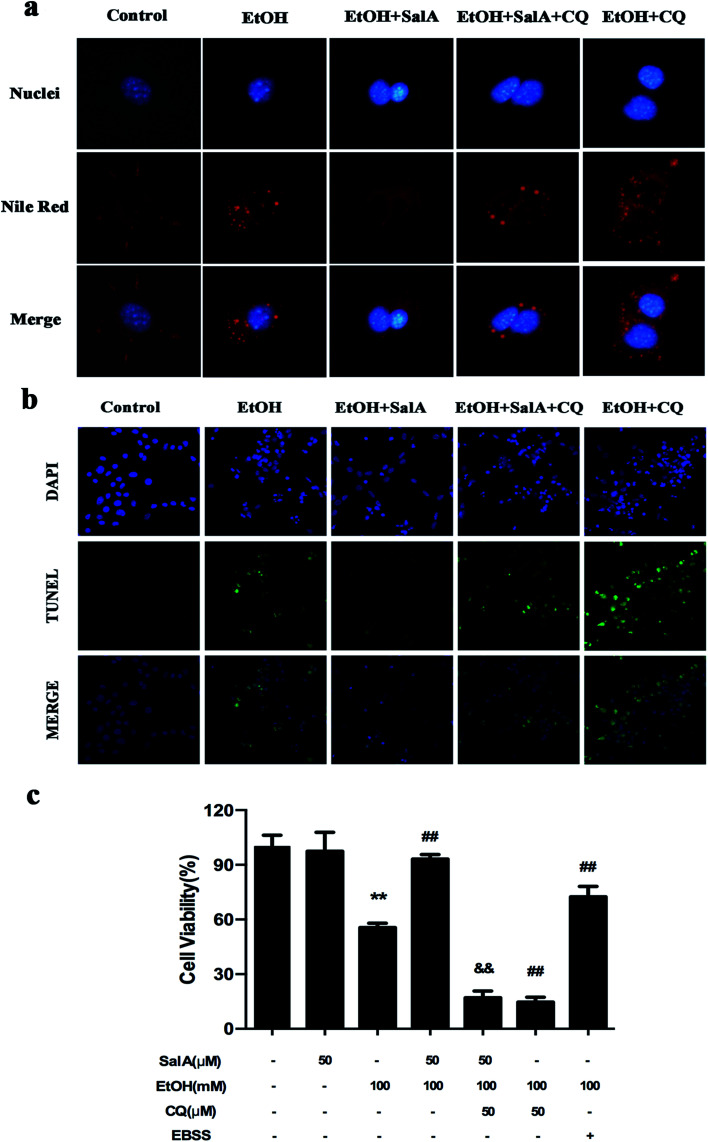
Autophagy activation by SalA exerts a protective effect against chronic ethanol-induced hepatocyte injury. (a and b) SalA-induced autophagy activation decreases lipid accumulation and apoptosis. AML-12 cells were pretreated with SalA (50 μM) or CQ (50 μM) for 6 h before exposure to ethanol (100 mM) for another 24 h. (a) Nile red staining (400×). (b) TUNEL staining (400×). (c) Cell viability. AML-12 cells were pretreated with SalA (50 μM), CQ (50 μM), or EBSS for 6 h and then treated with ethanol for 24 h; afterwards, cell viability was determined. The data are presented as the mean ± S.D. **P < 0.01 *versus* the control group, ^##^*P* < 0.01 *versus* the ethanol group, ^&&^*P* < 0.01 *versus* the ethanol + SalA group (*n* = 8).

The induction of autophagy can effectively alleviate alcoholic liver injury.^24^ Therefore, we investigated whether autophagy activation was associated with the protective effects of SalA against chronic ethanol-induced liver injury. A recent study demonstrated that excessive AV accumulation in hepatic steatosis does not represent autophagy activation but is a result of lipid overload-induced AV-lysosome fusion impairment.^36^ According to our results (Fig. 2a), after ethanol consumption, AVs were unambiguously observed by TEM. These findings suggest that chronic ethanol treatment induced AV accumulation in the liver. To study the entire autophagy process in the chronic ethanol model, we used adenoviruses to infect AML-12 cells and measure autophagy flux. Ethanol exposure increased AV formation, yet autolysosomes were rarely observed. These results indicate that chronic ethanol administration reduced autolysosome formation. However, the number of autolysosomes was increased by SalA pretreatment, which suggests that SalA can activate autophagy under conditions of chronic ethanol consumption (Fig. 2b). Next, we investigated whether SalA exerted this protective role by activating autophagy. It is well known that ethanol can lead to steatosis^6^ and hepatocyte apoptosis.^37^ Accordingly, lipid accumulation, apoptosis, and cell death were up-regulated by ethanol consumption, whereas SalA treatment considerably attenuated this process. Ethanol exposure aggravated lipid accumulation, apoptosis, and cell death in AML-12 cells treated with both SalA and the autophagy-specific inhibitor chloroquine (CQ) (Fig. 3a, b and c). The protective effects of autophagy during chronic ethanol consumption were further confirmed using the autophagy activator Earle's balanced salt solution (EBSS).^38^ SalA and EBSS treatment reversed ethanol-induced cell injury (Fig. 3c). These results indicate that activating autophagy by SalA could attenuate chronic ethanol-induced liver injury.

### SalA activates autophagy which is inhibited by chronic ethanol consumption *via* restoring AV-lysosome fusion

**Fig. 4:**
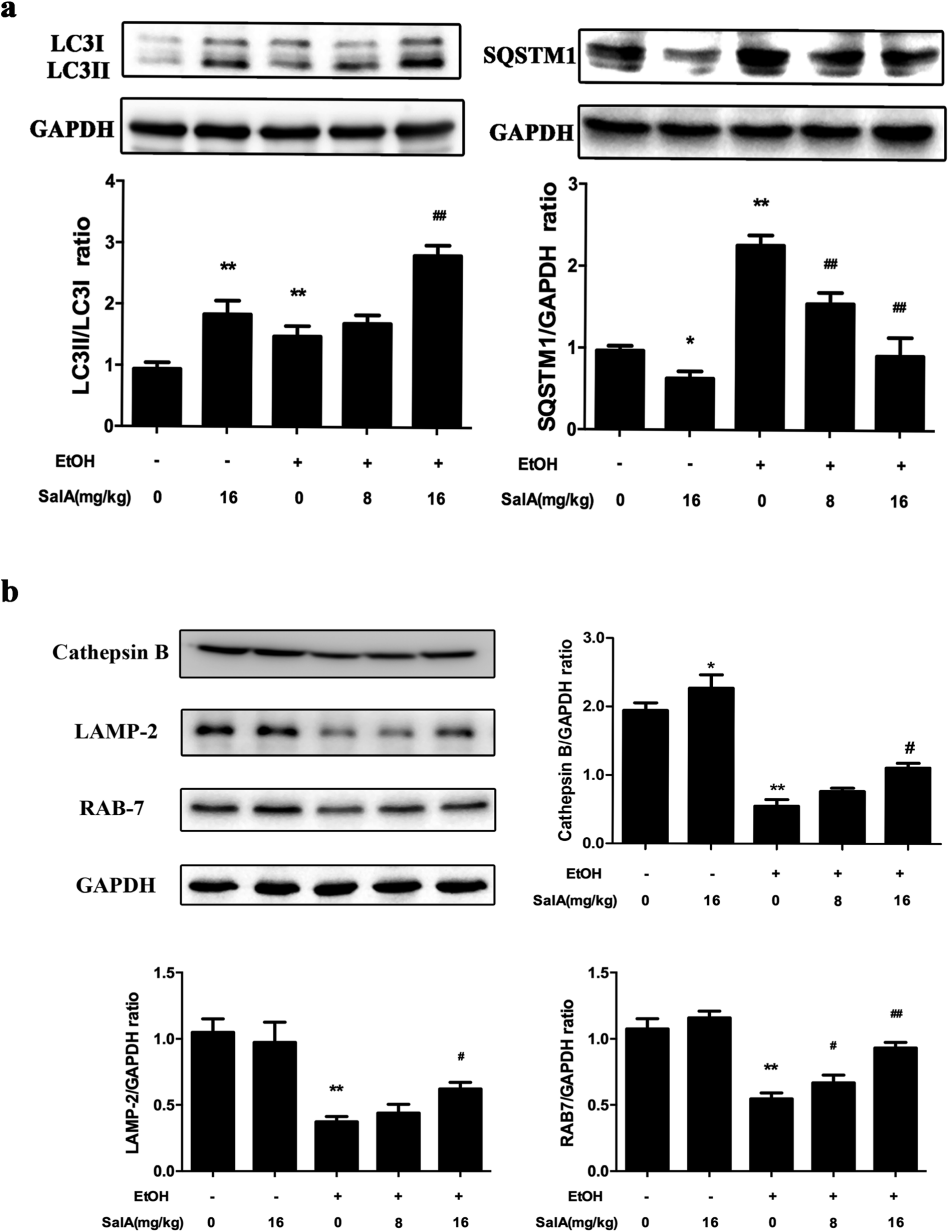
SalA promotes autophagy activation to prevent chronic ethanol-induced AV-lysosome fusion damage. (a and b) The expression levels of LC3-II, SQSTM1, the mature form of cathepsin B, RAB7, and LAMP-2 in the liver were evaluated by western blotting. The data are presented as the mean ± S.D. (*n* = 3). *P < 0.05 *versus* the ND group, **P < 0.01 *versus* the ND group, ^#^*P* < 0.05 *versus* the ED group, ^##^*P* < 0.01 *versus* the ED group.

To further assess whether SalA exerted its protective effects against ALD by regulating autophagy, we measured the expression levels of LC3-II and SQSTM1, which are marker proteins of autophagy and degradation in the lysosomes.^39,40^ The protein levels of LC3-II and SQSTM1 were higher in the ethanol group than those in the control group (Fig. 4a); however, LC3-II levels were even higher and SQSTM1 levels were lower in the SalA group than those in the ethanol group. Furthermore, we measured the protein levels of lysosomal proteins, including the mature form of cathepsin B, LAMP-2, and RAB7 (Fig. 4b). After chronic ethanol consumption, LAMP-2, RAB7 and the mature form of cathepsin B levels were decreased. However, pretreatment with SalA prior to ethanol exposure resulted in increased levels of LAMP-2, RAB7, and cathepsin B. Thus, the variable expression levels of autophagy-related proteins indicate that chronic ethanol consumption damaged AV-lysosome fusion and that SalA pretreatment could restore autolysosome formation and activate autophagy.

### SalA promotes AV-lysosome fusion in a SIRT1-dependent manner

**Fig. 5:**
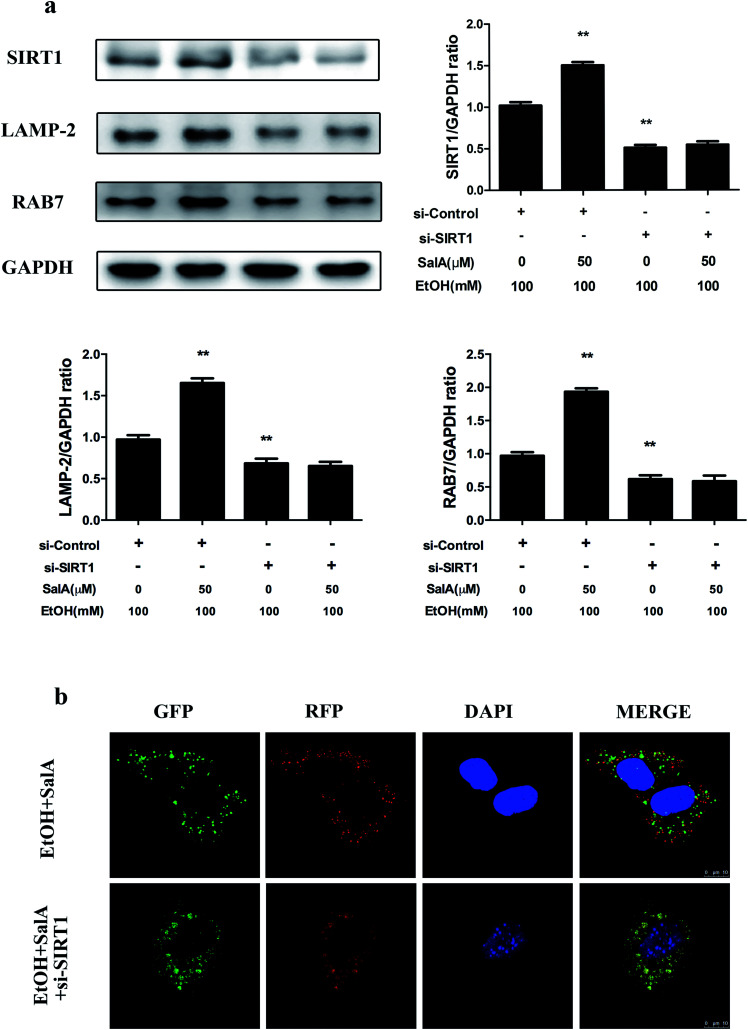
SIRT1 mediates the restoration of AV-lysosome fusion by SalA in ethanol-treated AML-12 cells. (a) AML-12 cells were transfected with SIRT1-specific siRNA or control siRNA for 48 h before treatment with SalA (50 μM). Then, the transfected cells were exposed to ethanol (100 mM) for another 24 h. SIRT1, LAMP-2, and RAB7 protein levels in cellular lysates were evaluated by western blotting. The data are presented as the mean ± S.D. **P < 0.01 *versus* the si-control + ethanol group (*n* = 3). (b) Autophagy flux visualized using confocal microscopy. AML-12 cells were infected with adenoviruses as described in Materials and methods.

SIRT1 is an NAD^+^-dependent protein deacetylase that regulates autophagy by modifying histones and transcription factors to activate autophagy flux.^31,33^ We thus examined whether the SalA-mediated promotion of AV-lysosome fusion in response to chronic ethanol exposure was regulated by SIRT1. As shown in Fig. 5a, compared with ethanol treatment alone, SalA pretreatment increased the SIRT1, RAB7, and LAMP-2 expression levels remarkably. We then evaluated the effects of SalA on AV-lysosome fusion after SIRT1 siRNA treatment in AML-12 cells. Compared with the control siRNA, SIRT1 siRNA treatment resulted in significantly decreased levels of RAB7 and LAMP-2 (ESI Fig. 1†). After chronic ethanol consumption, the SalA-induced increased levels of RAB7 and LAMP-2 were mostly abolished in the presence of SIRT1 siRNA (Fig. 5a). In addition, SIRT1 siRNA abrogated the SalA-induced activation of AV-lysosome fusion (Fig. 5b). These data suggest that SalA-mediated SIRT1 induction activated autophagy by promoting AV-lysosome fusion.

## Discussion

Alcohol-induced liver injury has become a worldwide disease. Although many treatments for ALD are currently being used, few are very effective. Thus, novel therapeutic approaches are required. The present research aimed to determine (1) whether SalA conferred protection against chronic ethanol-induced liver injury and (2) whether the protective effects of SalA against ALD were associated with autophagy activation caused by SIRT1-induced AV-lysosome fusion.

SalA is one of the primary active components that can be isolated from *Salvia miltiorrhiza*. Studies have reported that SalA exerts protective effects on the liver; it can prevent HFD-induced hepatic fibrosis, scavenge ROS, and protect against concanavalin A (ConA)-induced hepatitis.^13,15,41^ Our study aimed to demonstrate that SalA plays a protective role in chronic ethanol-induced liver injury. As expected, SalA protected the liver from chronic ethanol exposure, decreased high serum transaminase levels, attenuated histological liver damage, and prevented ethanol-induced liver cell injury *in vitro* (Tables 1, 2, and Fig. 1). We further explored the molecular mechanisms involved in the protective effects of SalA against ALD.

Studies have demonstrated that autophagy is an essential catabolic pathway that degrades cellular components within the lysosome. Moreover, autophagy is important for maintaining cellular functions and controlling lipid metabolism in the liver. Interestingly, chronic alcohol consumption suppresses autophagy; this finding indicates that decreased autophagy may underlie the pathogenesis of ALD.^42^ Alcohol induces AV formation but decreases the number of autolysosomes.^43,44^ These data are consistent with our results. However, SalA treatment markedly reversed this trend. This finding suggests that SalA may have the same effects as an autophagy activator in protecting against ALD (Fig. 2). We tested this possibility in the AML-12 cell line. As expected, SalA increased cell viability and elicited the same effects as EBSS (a specific activator of autophagy) after ethanol exposure (Fig. 3c). Ample evidence has indicated that the important role of autophagy in reducing ALD is likely due to the selective removal of lipid droplets and damaged mitochondria.^24,45,46^ Similarly, genetically increasing autophagy by overexpressing the autophagy gene Atg7 ameliorated hepatic steatosis and insulin resistance in ob/ob and HFD-fed mice.^47^ Growing research also indicates that the cytoprotective function of autophagy is mediated in many circumstances by the negative modulation of apoptosis. Under certain circumstances, autophagy constitutes a stress adaptation that suppresses apoptosis by regulating the BCl2-family proteins BAX and BAK and caspases to attenuate diseases such as Alzheimer's disease and hepatocellular carcinoma.^48–50^ In ALD, the activation of autophagy alleviates ethanol-induced apoptosis mediated by the AMPK/FoxO3A axis.^51^ In our study, the lipid accumulation and apoptosis induced by ethanol were diminished by SalA; however, SalA pretreatment was no longer protective after treatment with the autophagy inhibitor CQ (Fig. 3a and b). Collectively, these results indicate that SalA plays a protective role in chronic ethanol-induced liver injury *via* activating autophagy.

To investigate the protective role of SalA in chronic ethanol-induced autophagy inhibition, we measured the protein levels of LC3-II and SQSTM1. The lipidation of LC3-I to LC3-II is a very important step for AV development and maturation.^20^ However, the accumulation of LC3-II could represent either the induction of autophagy or the impairment of autophagy flux by blocking lysosomal fusion and degradation.^40^ Consistent with these findings, chronic ethanol or SalA treatment increased the expression levels of LC3-II. These data indicate that chronic ethanol or SalA treatment induced AV formation. However, increased expression levels of the autophagy substrate SQSTM1 suggest that chronic ethanol consumption inhibited the degradation of SQSTM1, whereas SalA pretreatment rescued this process (Fig. 4a). These results demonstrate that SalA pretreatment induced AV formation and the degradation of the autophagy substrate. Furthermore, we assessed whether SalA activated autophagy by promoting AV-lysosome fusion. Recent findings have suggested that chronic ethanol consumption diminishes the proteolytic capacity of liver lysosomes, decreases cathepsin B levels, blocks AV-lysosome fusion, and inhibits protein degradation.^23,40,52^ Previous reports have indicated that knocking down the lysosomal markers RAB7 and LAMP-2 inhibits AV-lysosome fusion;^33,53,54^ in addition, RAB7 is a central regulator that is important for regulating hepatic lipid droplet (LD)-specific autophagy (lipophagy).^55,56^ As expected, chronic ethanol exposure significantly decreased the levels of LAMP-2, RAB7, and the mature form of cathepsin B. These results indicate that AV-lysosome fusion was impaired, but pretreatment or co-treatment of SalA could both prevented these impairments (Fig. 4b, ESI Fig. 2†), consistent with the protective effect of Torin1, a known autophagy inducer^57^ (ESI Fig. 3†). Therefore, variation in the levels of these autophagy-related proteins illustrates that restoring AV-lysosome fusion and autophagy were required for SalA to alleviate chronic ethanol-induced liver injury.

SIRT1, an NAD^+^-dependent deacetylase, has important effects on metabolic diseases, including ALD. Chronic ethanol accumulation decreases SIRT1 expression levels, whereas SIRT1-specific transgenic mice are protected from hepatic steatosis caused by ethanol consumption.^58^ In our study, SIRT1 protein levels were significantly decreased after chronic ethanol exposure, whereas SalA treatment prevented this effect. These results suggest that SalA may confer protection against chronic ethanol-induced ALD by increasing SIRT1 expression (ESI Fig. 4 and 5a†). SIRT1 is well recognized as a primary regulator of autophagy through its deacetylation and activation of major components of the autophagy induction network; these components include ATGs and transcriptional regulators, such as ATG5, ATG7, CREB, and PPARα.^31^ Additionally, SIRT1 mediates the effect of adipose triglyceride lipase (ATGL) in promoting lipophagy to control hepatic lipid metabolism.^59^ Moreover, autophagy flux is damaged, and autophagy is inhibited in liver-specific SIRT1^−/−^ mice.^32,60^ A recent study revealed that SIRT1 protects against ischemia/reperfusion injury in alcoholic fatty liver through the activation of autophagy.^61^ However, this study did not determine whether SIRT1 regulated AV-lysosome fusion. We found that after chronic ethanol consumption, SIRT1 up-regulation *via* SalA increased LAMP-2 and RAB7 protein levels in AML-12 cells; however, SIRT1 knockdown notably inhibited this increase, suggesting that autophagosome–lysosome fusion promoted by SalA was mostly abrogated in the presence of SIRT1 siRNA (Fig. 5a). Furthermore, by using confocal microscopy, we found that SIRT1 knockdown increased the number of AVs while decreasing the number of autolysosomes; SalA treatment, however, could not reverse this trend (Fig. 5b). Together, these results suggest that the SalA-mediated recovery of AV-lysosome fusion and autophagy is regulated by SIRT1.

## Conclusions

In summary, the present study revealed that SalA had a protective effect against chronic ethanol-induced liver injury. The protective effect of SalA was associated with autophagy activation. The results indicate that SalA conferred protection against chronic ethanol-induced ALD, at least partly through the SIRT1-mediated promotion of AV-lysosome fusion and autophagy. Our study presents an attractive pharmacological target for the development of new drugs to prevent ALD progression.

## Conflicts of interest

There are no conflicts to declare.

## Supplementary Material

RA-008-C8RA00798E-s001
