# Supplementary material for: Promotion of autophagosome–lysosome fusion via salvianolic acid A-mediated SIRT1 up-regulation ameliorates alcoholic liver disease
Source: RSC Adv. 2018 Jun 5;8(36):20411–22. doi: 10.1039/c8ra00798e (PMC9080827; doi:10.1039/c8ra00798e)
Supplement: RA-008-C8RA00798E-s001 [file RA-008-C8RA00798E-s001.pdf]

Electronic Supplementary Material (ESI) for RSC Advances.
This journal is © The Royal Society of Chemistry 2018

Supplementary Fig. 1 siRNA-mediated the knockdown of SIRT1 inhibits the fusion of AV-lysosome. SIRT1, LAMP-2, and RAB7 protein expression levels in AML-12 cells were evaluated by Western blotting after transfection with a negative control or SIRT1-specific siRNA. The results are expressed as the mean±S.D. $^{**}P<0.01$ versus the control group (n=3).

Supplementary Fig. 2 SalA and chronic ethanol co-treatment increase the expressions of the lysosomal marker proteins. AML-12 cells were treated with or without SalA (50 μM) and ethanol (100 mM) for 24 h. The protein levels of LAMP-2 and RAB7 were evaluated by Western blotting. $^{*}P<0.05$ versus the control group, $^{***}P<0.001$ versus the control group, $^{\#\#}P<0.01$ versus the ethanol group (n=3).

Supplementary Fig. 3 SalA or Torin1 induces the activation of autophagy. AML-12 cells were pretreated with SalA (50 μM) or Torin1 (100 nM) for 6 h and then exposed to ethanol (100 mM) for 24 h. The protein levels of LAMP-2 and RAB7 were evaluated by Western blotting. $^{**}P<0.01$ versus the control group, $^{\#}P<0.05$ versus the ethanol group (n=3).

Supplementary Fig. 4 SalA regulates SIRT1 expression. AML-12 cells were incubated with or without 50 μM SalA for 6 h and then with or without 100 mM ethanol for 24 h. The SIRT1 protein level in cellular lysates was analyzed by Western blotting. $^{*}P<0.05$ versus the control group, $^{**}P<0.01$ versus the control

group, #P<0.05 versus the ethanol group (n=3).

Supplementary Figure 1

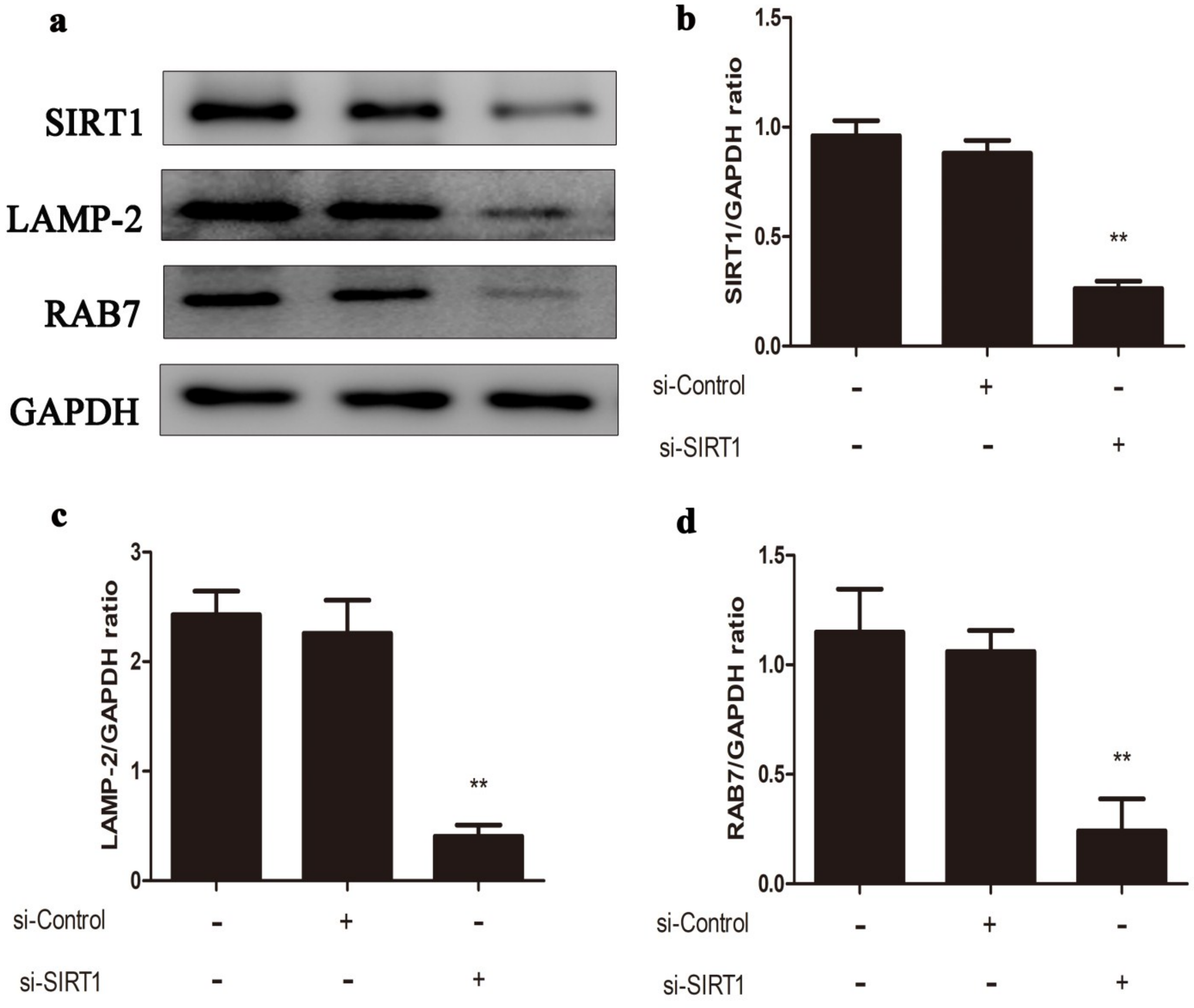

Fig S1.

Supplementary Fig. 2

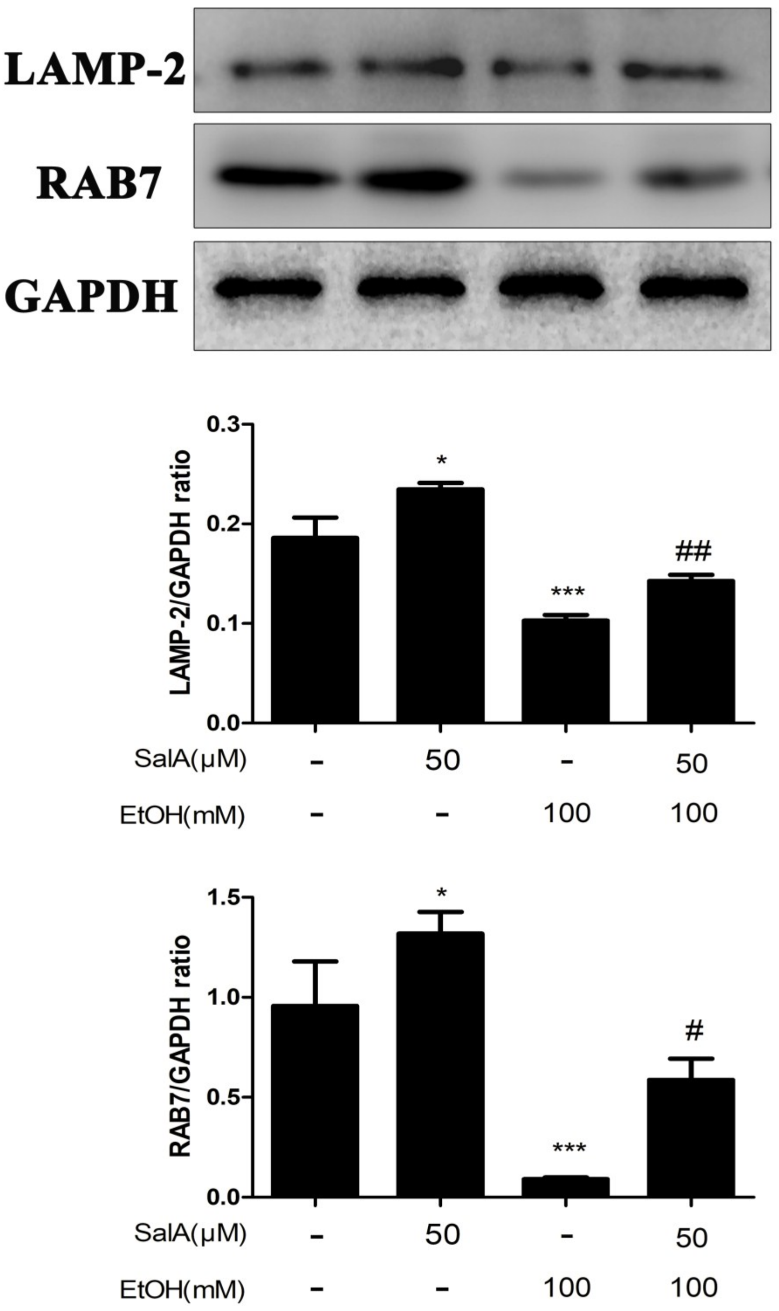

Fig S2.

Supplementary Fig. 3

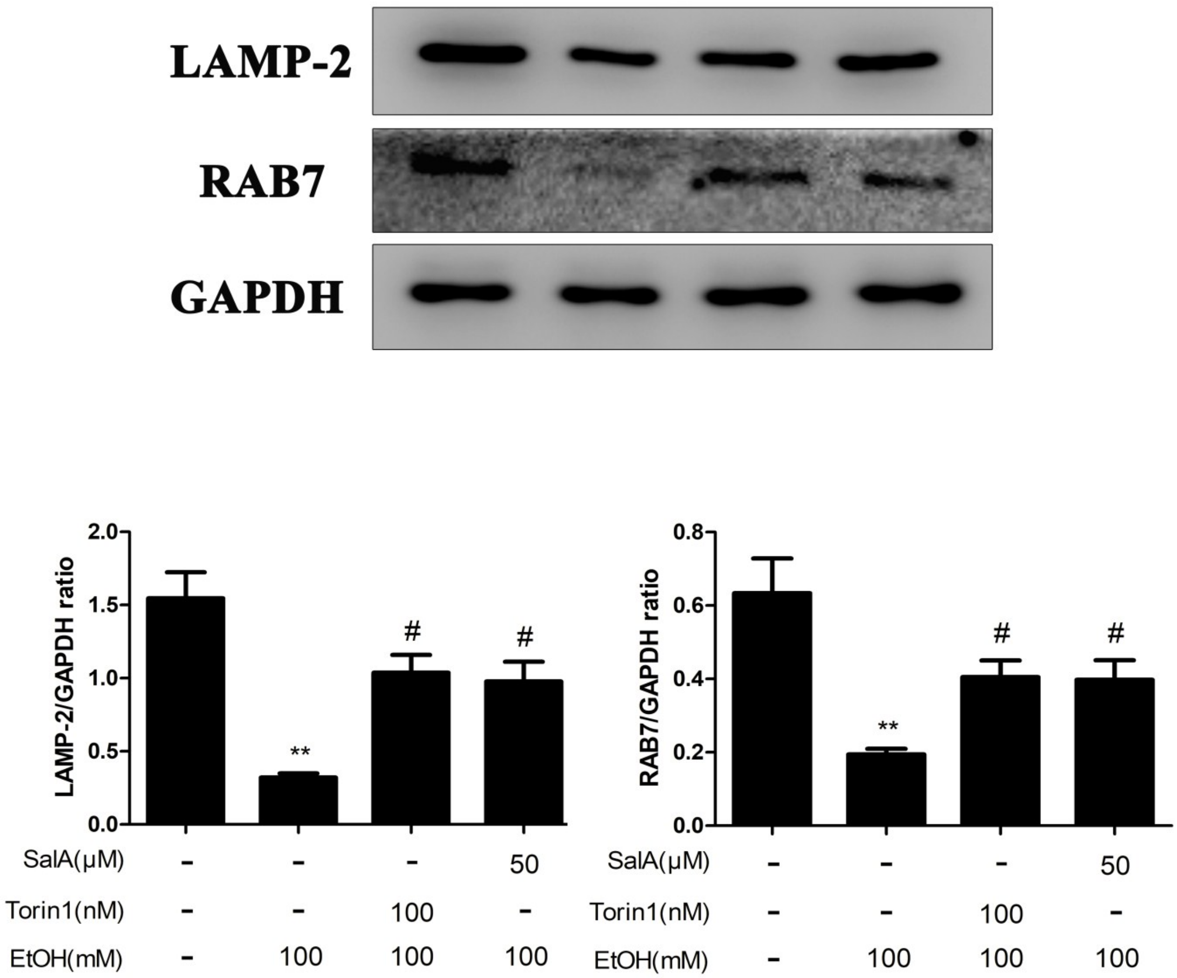

Fig S3.

Supplementary Fig. 4

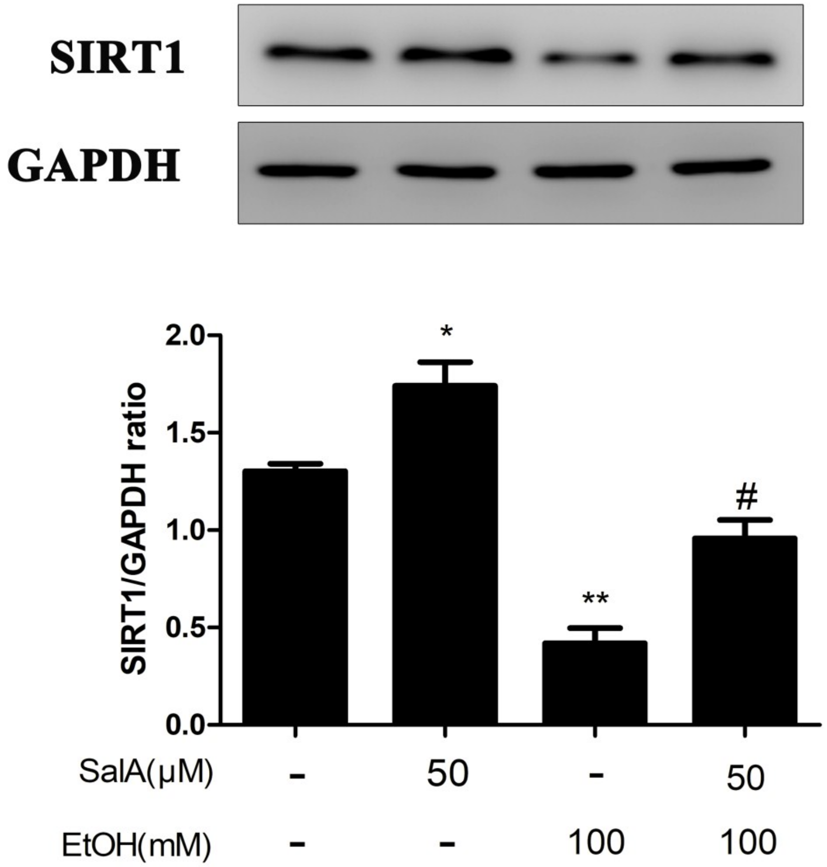

Fig S4.
